# The Evolution of Per-cell Organelle Number

**DOI:** 10.3389/fcell.2016.00085

**Published:** 2016-08-18

**Authors:** Logan W. Cole

**Affiliations:** Department of Biology, Indiana UniversityBloomington, IN, USA

**Keywords:** plastid, mitochondria, stoichiometry, organelle biogenesis, evolutionary cell biology

## Abstract

Organelles with their own distinct genomes, such as plastids and mitochondria, are found in most eukaryotic cells. As these organelles and their host cells have evolved, the partitioning of metabolic processes and the encoding of interacting gene products have created an obligate codependence. This relationship has played a role in shaping the number of organelles in cells through evolution. Factors such as stochastic evolutionary forces acting on genes involved in organelle biogenesis, organelle–nuclear gene interactions, and physical limitations may, to varying degrees, dictate the selective constraint that per-cell organelle number is under. In particular, coordination between nuclear and organellar gene expression may be important in maintaining gene product stoichiometry, which may have a significant role in constraining the evolution of this trait.

## Introduction

With a few exceptions (Cavalier-Smith, 1987; Karnkowska et al., 2016), mitochondria or plastids are found in all eukaryotic cells. As these organelles and their host cells have evolved together, the partitioning of metabolic processes and the encoding of interacting gene products have created an obligate codependence. This relationship has obviously played an important role in shaping the genomes of these different compartments (Howe et al., 2002; Adams and Palmer, 2003), but what has not been widely considered is how this intimate relationship has constrained the number of organelles in a cell through evolution.

Typically, a multicellular eukaryote has hundreds or thousands of these organelles in each of their cells. These numbers not only vary among species but also among tissue types (Veltri et al., 1990; Li et al., 2001; Moller, 2004) and temporally during the cell cycle, across development, and in response to stress (Holloszy, 1967; Posakony et al., 1977; Boffey and Leech, 1982; Visser et al., 1995; Moller, 2004; Kowald and Kirkwood, 2011). These organelles serve necessary metabolic functions and create gene products that are necessary not only for their own function but for that of the entire cell. This, along with the transfer of genes from the organelle to nuclear genomes over evolutionary time (Blanchard and Schmidt, 1995; Howe et al., 2002; Adams and Palmer, 2003), has created a necessary coordination between these entities. These factors, to some extent, have constrained the number of organelles in a cell through selection. Other factors, such as stochastic evolutionary forces, which vary depending on the population genetic environment of the genes involved in organelle biogenesis and organelle–nuclear interactions, or physical limitations may obscure the selective constraint that this feature is under.

## Estimating organelle number across the eukaryotes

Organelles can be counted directly using molecular staining (Williamson and Fennell, 1979; Chazotte, 2011) and microscopy, but this is very labor intensive. Alternatively, organelles can be quantified with some uncertainty through flow cytometry (Mattiasson, 2004), which has the drawback of underestimation that results from the isolation process, or through the use of biochemical probes (Robin and Wong, 1988). Recently, some have been using micro-dissection to estimate organelle number by extrapolating counts from cross sections (Inuda and Wildermuth, 2004; Kubinova et al., 2014). Some would argue that the methods that involve direct counting are the only ones appropriate to properly estimate the number of organelles in a cell, but they are impractical when it comes to some cases, such as animal cells, that have thousands of mitochondria.

These techniques are useful for low error quantification of organelle number within a cell but, with the advent of sequencing technology, one can use organelle genome copy number to estimate organelle number (Moraes, 2001). This, of course, comes with the consideration that there tends to be between 1 and 10 copies of mtDNA per mitochondrion in animals (Satoh and Kuroiwa, 1991; Weisner et al., 1992; Bereiter-Hahn and Voth, 1996; Iborra et al., 2004; Legros et al., 2004; Brown et al., 2011; Kukat et al., 2011), between 50 and 200 copies of mtDNA in yeast (Williamson and Fennell, 1979; Azpiroz and Butow, 1993; Solieri, 2010), and between 0 and 2 copies of mtDNA in plants (Suyama and Bonner, 1966; Bendich and Gauriloff, 1984; Preuten et al., 2010), though this estimate may be influenced by the presence of substoichiometric mtDNA molecules (Palmer and Shields, 1984; Preuten et al., 2010; Mower et al., 2012), and up to ~1000 copies of cpDNA per mature chloroplast (Boffey and Leech, 1982). These per-organelle genome copy numbers can also vary temporally; they can either change over the life span of the organelle as is the case with most chloroplasts (Lamppa et al., 1980; Scott and Possingham, 1980, 1983; Baumgartner et al., 1989; Kuroiwa, 1991), the lifespan of the organism as is the case with some mitochondria (Hartmann et al., 2011) and chloroplasts (Zoschke et al., 2007), or change erratically and unpredictably due to the unequal redistribution of nucleoid material between organelles during fusion and fission, which has been shown to be the case in some mitochondria (Arimura et al., 2004a).

A major caveat with these methods of quantification is that they tend to estimate organelle number at a particular time point or over a very small time range, while it is known that the number of organelles is a very labile trait that can vary a great deal over time (Stoecker and Silver, 1990; Arimura et al., 2004a).

Like many unicellular eukaryotes, which can have as few as a single mitochondrion or a few dozen mitochondria per-cell (Gray et al., 2004) and can also have on the order of 10^5^ mitochondria (Okie et al., 2016), multicellular eukaryotes tend to have a wide range of per-cell mitochondria numbers with estimates in mammalian somatic cells ranging from ~80 to ~2000 (Robin and Wong, 1988; Bogenhagen, 2011; Kukat et al., 2011) and from ~200 to ~600 in plant mesophyll cells (Logan, 2007). Yeast have been shown to vary in terms of per-cell mitochondria number based on substrate despite relatively consistent per-cell mtDNA copy number—for example, yeast grown on a glucose substrate were shown to contain 2–3 mitochondria that are branched in structure while those grown on an ethanol substrate contain 20–30 tubular-shaped mitochondria (Visser et al., 1995). Occasionally, there can be tens of thousands (Satoh and Kuroiwa, 1991) or even millions of mitochondria in an animal oocytes, as is the case in the oocytes of *Xenopus laevis*, which have been estimated to have on the order of 10^7^ mitochondria (Marinos, 1985). Mitochondria are very dynamic entities that are subject to frequent fusion and fission (Arimura et al., 2004a; Kowald and Kirkwood, 2011) so this can change over time. Both mitochondria (Gurdon et al., 2016) and chloroplasts (Thyssen et al., 2012) have been shown to move between cells in plants, even among those of different species, which may have some effect on per-cell counts of organelles. Some unicellular algae will tend to have a single chloroplast (Itoh et al., 1996) whereas some cells from plants, such as mesophyll cells, have been seen to have an average of ~50 chloroplasts at their earliest stages of development (Boffey and Leech, 1982) and up to hundreds of chloroplasts (Moller, 2004) with an average of 155 at their latest stages of development (Boffey and Leech, 1982). Similarly, mitochondria number has been shown to fall 25–50% during leaf senescence in *Vitis vinifera* (Ruberti et al., 2014). These numbers suggest that those early eukaryotes had one or a few of these organelles, as in some modern unicellular eukaryotes, and then underwent an increase followed by a diversification in organelle number, resulting in the variation in organelle number we see among multicellular eukaryotes.

These numbers not only vary significantly across organisms, but among tissue types. For example, there are more chloroplasts in leaf tissue than in other tissue (Li et al., 2001). Moist, leafy tissue in plants has been shown to have more mitochondria than woody and stem tissue (Moller, 2004). This is the result of leafy, green tissue being the important focal tissue of photosynthesis. Mesophyll and stomata cells have also been shown to lose mitochondria at different rates during leaf senescence (Ruberti et al., 2014). Studies of the mouse have shown that tissue of the liver, kidney, heart, and brain have different numbers of mitochondria per-cell (Veltri et al., 1990). Organelle number also varies in in terms of lability across tissue types. For example, muscle cells are known to vary greatly in mitochondrial content with organismal physical activity (Holloszy, 1967). The differences in mitochondria number among tissue types may be the result of varying energetic constraints (Holloszy, 1967; Veltri et al., 1990). It has also been shown that there can be large reductions in chloroplast number in stressful high and low light conditions in a variety of green plants (Higa and Wada, 2016).

The extent to which per-cell mitochondria number varies within tissue is largely unknown. Many methods used to count mitochondria within multicellular eukaryotes involve large samples of tissue with flow cytometry or other methods that represent an aggregate of many cells within a tissue (Mattiasson, 2004). *In vivo* estimates would require microscopy of many cells within the same tissue of the same organism. Variability has been assessed within a HeLa cell line, which was shown to contain between 383 and 882 mitochondria per-cell (Posakony et al., 1977), though this is mostly the result of differences in cell cycle stages. Furthermore, this should not be interpreted as a reflection of natural or *in vivo* variation of within-tissue organelle number. Within-tissue variation in chloroplast number, however, has been shown to be rather extensive with between ~2 and ~140 fold variation in chloroplast number in palisade tissue from different green plants (Higa and Wada, 2016). To what extent and how within-tissue per-cell organelle number variation is maintained will require more data and this will serve as a prerequisite to analyses with methods from quantitative genetics, developmental biology, and physiology.

## Organelle biogenesis

At the molecular level, per-cell organelle number is underpinned by series of transcriptional pathways. These processes are distinct but involve related components in chloroplasts and mitochondria that mostly control the pattern of division of organelles and the replication and transcription of their genomes. An understanding of what genes are involved in organelle biogenesis is necessary to fully understand the evolution of organelle number in cells.

The so-called “master regulators” of mitochondrial biogenesis are members of the PGC (peroxisome proliferator-activated receptor-γ) family of co-transcriptional regulatory factors (Irrcher et al., 2003; Ventura-Clapier et al., 2008). In particular, PGC-1α is known to activate different transcription factors that interact with Tfam (mitochondrial transcription factor A), which is involved in the replication and transcription of mtDNA and the transcription of nuclear-encoded mitochondrial components (Virbasius and Scarpulla, 1994). Though the connection between PGC-1α and the physical division of mitochondria is not fully understood, it has been shown to increase respiration in cells (Wu et al., 1999). The division of mitochondria is known to involve the dynamin-like proteins (Arimura et al., 2004b) and Fis-type proteins (Lu et al., 2011). These pathways are mostly understood from the perspective of human and mouse mitochondria, so these processes may be quite different in the mitochondria of other eukaryotes.

Plastid biogenesis is much less well-understood than mitochondrial biogenesis because many factors implicated in this process are often only known from the phenotyping of *Arabidopsis* mutants (López-Juez, 2007). Those factors whose functions are well-understood seem to mostly be involved in the formation of plastid division rings including dynamin-like proteins (Gao et al., 2003), like those involved in mitochondrial biogenesis. As such, the process of plastid division is mostly understood to be a mechanical one with daughter plastid components mostly being the result of random, unequal segregation of the contents of the parent cell (López-Juez, 2007), though some components of the plastid, such as thykaloids, seem to have an equitable redistribution between daughter plastids as regulated through the ARTEMIS and PEND proteins (Fulgosi et al., 2002; Terasawa and Sato, 2005).

## The population genetic environment of genes involved in organelle number

The genes involved in determining organelle number mostly fall within one of two categories: (1) those that are in the nuclear genome, including those that control the division of organelles and the over two-hundred genes that encode products that interact with organelle-encoded proteins (van Wijk, 2004; Giegé et al., 2005; van Wijk and Baginsky, 2011) and (2) those that encode proteins in the organelle genomes themselves, especially those that interact with nuclear proteins, whose potential importance will be explained later in the essay. The three compartments in which these genes reside present different population genetic environments that presumably play a large role in dictating the relative power of selection in the evolution of organelle number.

Population mutation rate (often represented by π or θ), which is a reflection of the population genetic environment as influenced by mutation and drift, is 2*N*_g_*u* where *u* is the mutation rate per nucleotide per generation and *N*_g_ is the effective number of genes at a locus (Lynch, 2006). For diploid nuclear genomes, this term is equal to 4*N*_e_*u* where *N*_e_ is the effective population size. This is important to consider as genes that dictate organelle number, like those that regulate mitochondrial biogenesis such as the PGC family (Ventura-Clapier et al., 2008) and those that encode the dynamin-like proteins and Fis-type proteins that are involved in plastid and mitochondrial division (Gao et al., 2003; Arimura et al., 2004b; Lu et al., 2011), are encoded in the nucleus.

The argument has been made that, due to the haploidy and maternal transmission of organelles, population mutation rate is simply equal to *N*_e_*u* in organelle genomes (Palumbi et al., 2001). Alternatively, it has been argued that this idea assumes an incorrect parity of selection and recombination between nuclear and organelle genomes and equivalency between males and females with respect to progeny (Lynch et al., 2006). Furthermore, organelle mutation rate is thought to vary much between different taxa. For example, when compared to nuclear genomes, animal mitochondrial mutation rates appear to be much higher (Brown et al., 1979) and plant mitochondrial mutation rates appear to be much lower (Palmer and Herbon, 1988).

Regardless of any of these considerations, the effective population size is generally thought to be smaller in organelle genomes (Palumbi et al., 2001; Lynch et al., 2006) and, as such, the strength of drift is thought to be greater than that of the nucleus, but the efficacy of selection could be just as great (Cooper et al., 2015) and vary greatly between different taxa with different mutation (Brown et al., 1979; Palmer and Herbon, 1988) and recombination regimes (Rokas et al., 2003; McCauley, 2013). As a result of these differences, for example, if organelle-encoded genes are important in the determination of per-cell organelle number, the efficacy of selection on organelle number may be more so in animals than in plants because of the greater mutation rate (Brown et al., 1979; Palmer and Herbon, 1988) and lower incidence of recombination (McCauley, 2013) in animal mitochondrial genomes.

## Metabolism as a potential selective constraint

The metabolic needs of a cell and the capacity for organelles to fulfill these needs may act as selective constraints on the number of organelles in a cell. Mitochondria, for example, perform a few different metabolic processes, such as the production of ATP through primarily aerobic respiration, regulation of cellular metabolic processes (McBride et al., 2006), and steroid synthesis (Rossier, 2006). Chloroplasts perform photosynthesis, producing NADPH and ATP through light reactions and glucose through the Calvin cycle, and are also involved in fatty acid (Rawsthorne, 2002) and amino acid synthesis (Burgess, 1989).

Metabolic needs have been thought to drive the relationship between the total amount of mitochondria and body mass across organisms according to a power law (Kleiber, 1947). Per-cell mitochondria content is also seen to change with cell size across the cell cycle (Posakony et al., 1977). Single-celled eukaryotes do not appear to follow Kleiber's power law for mitochondria or chloroplasts, but do appear to follow linear and sublinear scaling, respectively, for organelle number with cell size (Okie et al., 2016). This study also shows that organelle size does not appear to scale strongly with cell size in single-celled eukaryotes, suggesting that per-cell organelle number is more important than organelle size as a means of modulating energetic requirements at the scale of the cell. Rafelski et al. (2012), however, did note a sublinear, positive correlation between cell volume and total organelle volume in yeast. Given the relationship between cell size and per-cell mitochondrial content, perhaps there is an optimal per-cell mitochondria number given cell size and the nature of mitochondrial biogenesis.

It may soon be possible to obtain an estimate of whole-cell energetic requirements per unit time and use this to determine the optimal per-cell mitochondrial content to further understand the role of selection on this trait through quantitative genetics. Though it is not yet possible to completely understand the energetic needs per unit time in a eukaryotic cell, there has been some progress in developing theoretical models that can estimate the metabolic and energetic needs of a single cell (Suthers et al., 2009; Karr et al., 2012; Lynch and Marinov, 2016).

## Coordination between the nucleus and organelles: transcription and translation

Since the original endosymbiosis events, there has been a substantial integration of the function of organelles and their hosts, some of which is due to the transfer of genes from organelle to nuclear genomic compartments over evolutionary time (Blanchard and Schmidt, 1995). As a result, most protein products encoded in the organelles form complexes with those that are encoded in the nucleus. There have also evolved to be nuclear-encoded transcription factors (Leigh-Brown et al., 2010) and proteins involved in post-transcriptional modification such as RNA editing (Schmitz-Linneweber and Small, 2008) that regulate the expression of mitochondrial genes. As such, some level of stoichiometric balance has to be maintained between nuclear- and organelle-encoded factors during the processes of transcription and translation to ensure the proper allocation of resources and to prevent waste in terms of energy and macromolecules. Maintenance of proper stoichiometric ratios for gene products is necessary, at least to some degree, in maintaining some semblance of physiological homeostasis at both the cellular and organismal levels.

Many mitochondrial encoded proteins (Giegé et al., 2005) and almost all plastid encoded proteins (van Wijk, 2004; van Wijk and Baginsky, 2011) form multi-subunit complexes with nuclear encoded products, of which there are about two-hundred. Among these complexes are ribosomes and ATP synthases of both organelles. Rubisco, PEP, and the major photosynthetic complexes of the plastid and all four major complexes of the electron transport chain in the mitochondrion are also among those formed by complexing with nuclear subunits. These are formed from specific stoichiometric ratios of subunits. The number of organelles could play a significant role in influencing the number of these products produced in a cell and, as such, the nuclear-organelle subunit stoichiometry could act as a significant constraint on the number of organelles in the cell.

Though levels of transcription do differ between genes, mitochondrial transcription in animals is mostly performed at a constitutive level (Bendich, 1988) because it occurs over the entire circular mitochondrial genome, one strand at a time (Lee and Clayton, 1998). This constitutive transcription and the tenuous connection between metabolic activity and gene expression in mitochondrial genomes (Bendich, 1988) suggest that, in order to maintain the appropriate numbers of transcripts, other means at other levels must be utilized by the cell. Another potential means of controlling gene product stoichiometry may be by adjusting organelle DNA copy number per organelle, but it has been shown that, since this quantity varies so little (Boffey and Leech, 1982; Weisner et al., 1992), per-cell organelle number rather than per-organelle DNA copy number is the major driver of per-cell organelle DNA copy number (Robin and Wong, 1988; Zoschke et al., 2007). Adjusting the number of organelles could act as a broad-scale blunt means of regulation for these genes, as an alternative to regulating expression on a gene-by-gene or genome-by-genome basis. As such, per-cell organelle number could play an important role in maintaining the stoichiometry between nuclear- and organelle-encoded subunits.

Given a particular cell, there could be an optimal number of organelles to maintain the stoichiometry between nuclear and organelle gene products. Counting gene products can be performed at the level of transcription by looking at the number of particular mRNAs in a cell (Itzkovitz et al., 2012) or at the level of translation by counting the number of particular proteins in a cell (Huang et al., 2004). By doing this, we could see how closely these quantities track the expected stoichiometry in some cases. Since we can estimate the number of organelles in a cell by the methods stated earlier, it may be possible to survey many cells of the same type for the density of organelles and see if there is an optimum that gives a ratio of gene products closest to the expected stoichiometric ratio.

If we can make the assumption that it is most fit for cells to produce the least amount of excess gene product for those proteins that form obligatory complexes, looking at many cells may be able to tell us which have the closest to the optimum per-cell organelle number. A comparison of the typical per-cell organelle density to our best estimate of the optimum per-cell organelle density may tell about the role of evolution in shaping organelle number. For example, if most cells have a far-from-optimal organelle number per-cell, it may merit further investigation into the possibility of there being a drift barrier (Lynch, 2011) or some other, previously not understood, physical constraint that is preventing most cells from reaching this stoichiometric optimum.

## Conclusion

Metabolism and the production of organelle encoded gene products are both important to consider in the context of organelle and cell evolution. Cells have energetic requirements and need to maintain some semblance of particular gene product quantities in order to function properly. Work in the fields of biophysics and physiology will allow us to determine how these processes are impacting fitness and constraining per-cell organelle number. To what extent these processes constrain per-cell organelle number through selection will be more clear as the field of cell-scale proteomics becomes more empirically and computationally tractable. As these techniques become increasingly practical, we will be able to apply the analytical approaches of population and quantitative genetics to better understand this issue from an evolutionary perspective.

## Author contributions

The author confirms being the sole contributor of this work and approved it for publication.

### Conflict of interest statement

The author declares that the research was conducted in the absence of any commercial or financial relationships that could be construed as a potential conflict of interest.
